# Quantification of Surface GalNAc Ligands Decorating Nanostructured Lipid Carriers by UPLC-ELSD

**DOI:** 10.3390/ijms20225669

**Published:** 2019-11-12

**Authors:** Laura Gauthier, Mathieu Varache, Anne-Claude Couffin, Colette Lebrun, Pascale Delangle, Christelle Gateau, Isabelle Texier

**Affiliations:** 1 Université Grenoble Alpes, CEA, LETI-DTBS, F-38000 Grenoble, France; laura.gauthier@cea.fr (L.G.); mathieu.varache@googlemail.com (M.V.); accoufin@gmail.com (A.-C.C.); 2 Université Grenoble Alpes, CEA, CNRS, IRIG-SyMMES, F-38000 Grenoble, France; colette.lebrun@cea.fr (C.L.); pascale.delangle@cea.fr (P.D.)

**Keywords:** nanostructured lipid carriers, N-acetyl-D-galactosamine ligand, surface functionalization, ligand quantification

## Abstract

Nanoparticles have been extensively studied for drug delivery and targeting to specific organs. The functionalization of the nanoparticle surface by site-specific ligands (antibodies, peptides, saccharides) can ensure efficient recognition and binding with relevant biological targets. One of the main challenges in the development of these decorated nanocarriers is the accurate quantification of the amount of ligands on the nanoparticle surface. In this study, nanostructured lipid carriers (NLC) were functionalized with N-acetyl-D-galactosamine (GalNAc) units, known to target the asialoglycoprotein receptor (ASGPR). Different molar percentages of GalNAc-functionalized surfactant (0%, 2%, 5%, and 14%) were used in the formulation. Based on ultra-high-performance liquid chromatography separation and evaporative light-scattering detection (UPLC-ELSD), an analytical method was developed to specifically quantify the amount of GalNAc units present at the NLC surface. This method allowed the accurate quantification of GalNAc surfactant and therefore gave some insights into the structural parameters of these multivalent ligand systems. Our data show that the GalNAc decorated NLC possess large numbers of ligands at their surface and suitable distances between them for efficient multivalent interaction with the ASGPR, and therefore promising liver-targeting efficiency.

## 1. Introduction

Since the early 1990s, nanomedicine has emerged as one of the most exciting areas of medical research. Different types of nanoparticles, including inorganic and organic nanoparticles, present unique properties in terms of physical (optic, electronic, and/or magnetic), chemical, and biological properties that can be exploited for various medical applications such as therapy, diagnosis, drug delivery, and vaccine development [1,2]. To maximize therapeutic efficiency and minimize undesired side effects of active pharmaceutical ingredients (API), increasing attention is being paid to lipid nanoparticles. These nanocarriers, including lipid nano-emulsions, solid lipid nanoparticles, lipid nanocapsules and nanostructured lipid nanocarriers (NLC), can encapsulate active compounds, protect them from the degradation by the biological media and then maximize their bioavailability [3,4,5,6]. Due to their lipid nature, they offer further advantages in terms of biocompatibility, safety of used components, production cost and controlled release of the payload [7]. Moreover, the inherent properties of these nanocarriers affect the dose limitation of administrated compounds by modifying their biodistribution pattern, providing protection against clearance, and/or increasing their availability in targeted organs, tissues, or cells [3,4,8]. In this context, NLC (nanostructured lipid carriers) introduced by Muller in 2002 [5], are particularly attractive due to the absence of organic solvent for their preparation, their high stability in vivo and their highly efficient encapsulation of lipophilic compounds [3,5,9,10]. Recently, a new generation of NLC based on the use of FDA approved components has been developed by our team [11]. These nanoparticles hold great promise for in vivo imaging and drug delivery [12,13]. Their efficient encapsulation of lipophilic compounds as well as their long-life colloidal stability (>1 year for certain formulations) make them ideal candidates as drug delivery systems.

Recent advances in drug delivery suggest the modification of the surface of nanocarriers to affect their physicochemical properties and to ensure API supply at the targeted location. The most popular coating component extensively used in this context is polyethylene glycol (PEG) [14]. Attachment of PEG to the surface may reduce interactions with proteins and uptake in macrophages, resulting in increased blood circulation/retention time [14,15,16,17]. Such “passive” targeting can ensure the accumulation of the drug in disease locations, such as tumors, and then improve its therapeutic benefit.

To go further in specific API delivery at the required site, active targeting nanocarriers have been explored [18,19]. Numerous cell-specific receptors are involved in essential biological functions, including signaling, trafficking, and transducing, and are recognized by ligands, including proteins, peptides, antibodies, nucleic acids, vitamins, and carbohydrates. To actively target diseased tissues or organs, ligands known to specifically interact with cell-specific receptors may be attached at the surface of nanocarriers. Carbohydrates, including mono-, oligo-, and polysaccharides, possess key recognition roles, mainly at the surface of cells, in numerous biological processes [19]. For instance, monosaccharides exhibit specific binding/interaction to cell-specific receptors, such as C-type lectin receptors, expressed on the surface of various cell types. In particular, the asialoglycoprotein receptor (ASGPR), a C-type lectin mainly expressed on the surface of hepatic cells [20,21], recognizes specifically galactose (Gal) and N-Acetylgalactosamine (GalNac) with an affinity for GalNAc approximately 50-fold higher than for Gal [22,23,24]. This receptor has been extensively studied as a promising candidate for liver-targeted drug delivery [19,25]. To further improve the affinity of Gal or GalNAc monosaccharide toward this hepatic receptor, multivalent interactions, such as those offered by the surface of decorated nanocarriers, have proven to be a promising strategy to improve efficiency as well as selectivity of the recognition process [26,27,28,29,30,31,32,33]. In this multivalent approach, several structural parameters, such as the nature, number and spatial organization of the monosaccharide, play key roles in the recognition process through the ASGPR. In molecular systems, optimized recognition was achieved when monosaccharides were approximately 20 Å apart [30]. Furthermore, several Gal decorated lipid nanoparticles have shown higher uptake in hepatic cells than their non-functionalized counterparts [34,35,36,37].

One of the main challenges in the development of these targeted nanocarriers is the access to well-defined multivalent carbohydrate systems, which requires the precise quantification of the number of monosaccharide ligands actually present at the surface of the nanoparticles. This latter parameter is indeed very important to get a better understanding of the recognition processes between the functionalized nanocarriers and targeted receptors. In the few studies reporting Gal or GalNAc functionalized lipid nanoparticles [35,38], the authors assume the total incorporation of the saccharide moieties on the nanoparticle surface, and to our knowledge, the precise quantification of saccharide ligands at the surface of nanocarriers has never been performed.

In this work, nanostructured lipid carriers (NLC) were decorated with GalNAc monosaccharide units to target the ASGPR mainly expressed at the surface of liver cells. The NLC prepared using the ultrasonication technique were formulated using different initial amounts of GalNAc modified amphiphilic PEG-derived surfactants. To assess the exact amount of GalNAc incorporated and present at the NLC surface, a method leading to the quantification of PEG-modified molecules was developed based on ultra-high-performance liquid chromatography as a separative method and evaporative light-scattering detection for detection (UPLC-ELSD). 

## 2. Results

### 2.1. SA-PEG_100_-GalNAc 6 Synthesis

**Scheme 1:**
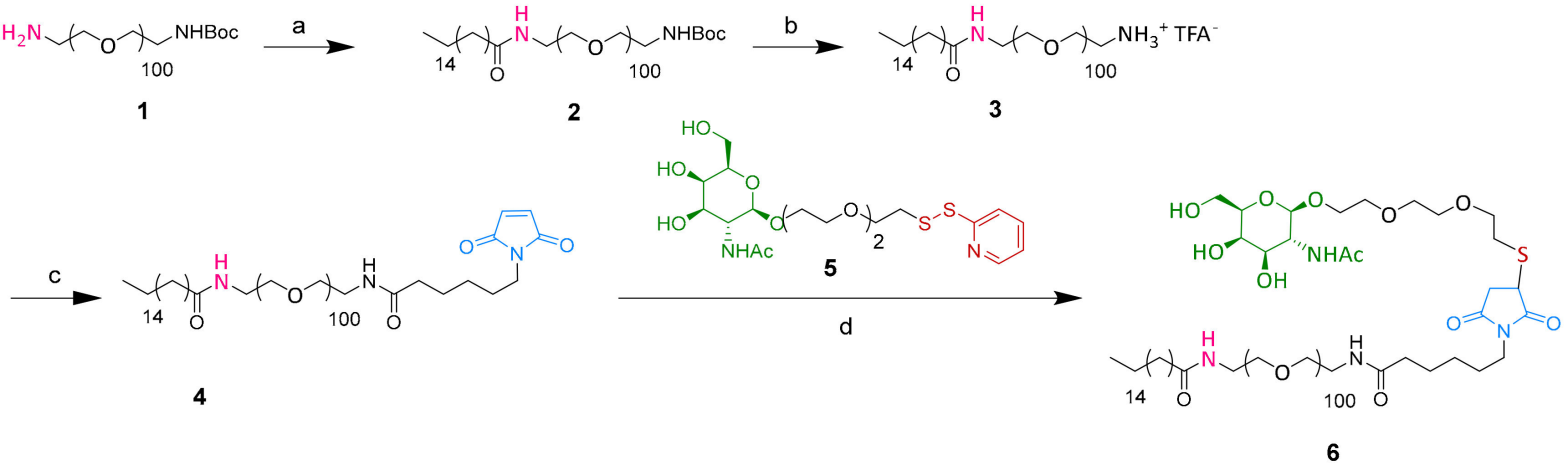
Synthesis of SA-PEG_100_-GalNAc 6 - Reactants and conditions: (**a**) Stearic acid, BOP, DIEA, DCM, 61%, (**b**) TFA, DCM, 85%, (**c**) 6-maleimidohexanoic acid, BOP, DIEA, DCM, 94%, (**d**) TCEP, ACN/H_2_O, 87%.

A novel N-acetyl-D-Galactosamine stearic acid (SA) PEG derivative (SA-PEG_100_-GalNAc **6**) was synthesized according to synthetic Scheme 1. This derivative presents bifunctional properties with a lipophilic anchor moiety (SA) for stable incorporation into the nanostructured lipid carriers and an N-acetyl-D-Galactosamine moiety for recognition by GalNAc specific receptors/transporters (e.g., asialoglycoproteins). The PEGylated chain of **6** comprised of about 100 ethylene oxide units, longer than that of Myrj^TM^S40 surfactant (about 40 ethylene oxide units) coating and stabilizing the NLC surface.

SA-PEG_100_-GalNAc **6** was obtained by grafting the synthetic GalNAc-SSPy derivative **5** and SA-PEG-maleimide **4** via a maleimide/thiol link (Scheme 1). The GalNAc-SSPy derivative **5** was synthesized according to a previously described procedure in four steps with an overall yield of 10% [33]. The stearate chain was grafted onto commercial NH_2_-PEG_100_-NHBoc using an amide bond using BOP as a coupling agent to give after deprotection the SA-PEG_100_-NH_3_^+^TFA^-^ **3** with an overall yield of 52% (Scheme 1). Then the maleimide group was grafted using BOP coupling. After the reduction of the disulfide bond present in the GalNAc-SSPy derivative **5** with TCEP, the free-thiol group reacted specifically with the maleimide function present on the SA-PEG_100_-maleimide **4** derivative to lead to the desired SA-PEG_100_-GalNAc compound **6** with an 87% yield. The reaction was followed by ^1^H NMR spectroscopy and stopped after the complete disappearance of the maleimide protons (6.7 ppm) (Supplementary Information, Figure S1).

### 2.2. Formulation of Nanostructured Lipid Carriers

NLC with and without different molar percentages of SA-PEG_100_-GalNAc **6** were formulated by ultrasonication according to previously described protocols [11]. Four different formulations were prepared with respectively 0% (Formulation A), 2.0% (Formulation B), 5.3% (Formulation C) and 13.6% (Formulation D) of SA-PEG_100_-GalNAc compared to Myrj^TM^ S40 (molar percentages). The ratios of Myrj^TM^ S40 and SA-PEG_100_-GalNAc varied while the total mass of PEGylated compounds, as well as the mass of other ingredients, were kept constant. Formulation E comprising 13.6% of Myrj^TM^ S100 (S100, long-chain PEG surfactant of around 100 ethylene oxide units) was also synthesized as a control. Component quantities are reported in Table 1.

Dynamic light scattering (DLS) was used to determine the NLC hydrodynamic diameter, polydispersity index (PDI), and zeta potential, as summarized in Table 2. Formulations A and B displayed similar hydrodynamic diameter and PDI, which were consistent with previously described studies [11]. Formulations C and D showed larger hydrodynamic diameter, and larger PDI for Formulation D. These differences may be due to the higher amount of SA-PEG_100_-GalNAc, which polymer chain presents a 2.5 fold larger number of ethylene oxide units than Myrj^TM^S40 [11]. This hypothesis was confirmed by data of Formulation E, comprising the same ratio of Myrj^TM^S100 long-chain surfactant. Zeta potential values were not significantly different: All nanoparticles presented surface charge close to neutrality, with a slightly negative value (presence of small quantities of hydrolyzed fatty acids in wax and oil components).

The long-term colloidal stability of formulations A–E was evaluated by the measurement of hydrodynamic diameter and PDI over storage time. As described previously [11], NLC issued from Formulation A were stable over one year (diameter increase < 10%, PDI < 0.2), whereas formulation stability decreased with increased content of SA-PEG_100_-GalNAc or Myrj™S100 (Table 2). Longer PEG_100_ chain could present different conformation than PEG_40_, modifying the surfactant organization in the nanoparticle shell that could account for the reduced oil/water stability leading to NLC destabilization. However, formulations B–E were stable enough to perform reliable further characterization experiments. 

### 2.3. Development of the Analytical Method for the Quantification of GalNAc Units Grafted on NLC Surface

#### 2.3.1. Optimization of the Analytical Conditions

**Figure 1:**
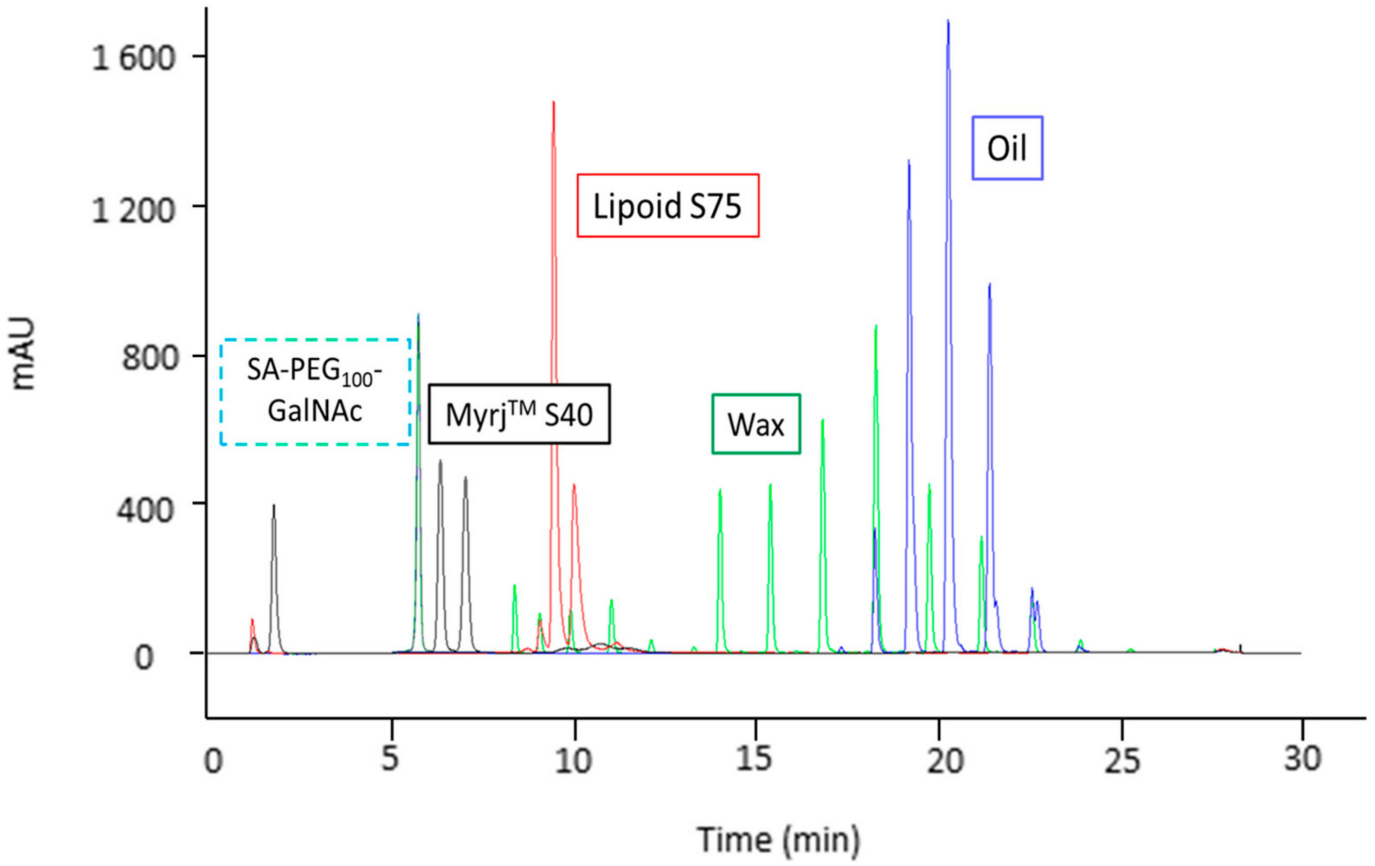
Combination of free nanostructured lipid carriers (NLC) component chromatograms (1 mg/mL for each compound).

To analyze each component of the formulations, NLC were disassembled by precipitating the lipids in acetonitrile. Therefore, components of NLC were first separately analyzed by UPLC-ELSD according to previously described analytical methods [39]. Briefly, two UPLC-ELSD methods were previously developed for the analytical qualification of NLC, one using a reverse-phase (RP) C18 column (method A), separating and quantifying Myrj^TM^ S40 and lipids, and the second one, a HILIC column (method B) for the assessment of lecithin components. Analytical method A, which comprised a two-step gradient elution program, succeeded in analyzing the exact content of Myrj™S40 (three compounds), soybean oil (15 compounds), and Suppocire™NB (15 compounds) [39]. To specifically analyze additional SA-PEG_100_-GalNAc, the proposed RP-UPLC-ELSD method was herein optimized by extending the first gradient step (from 3 to 15 min), thus allowing better separation of Myrj™S40 and SA-PEG_100_-GalNAc. The gradient elution program used in this study is reported in Table 3 (Materials and Methods). The chromatogram of all the NLC separated components are superimposed in Figure 1.

According to the supplier information, Myrj^TM^ S40 is a non-ionic surface active agent which was calculated to contain 71.3% (*w*/*w*) of three different compounds: PEG-C_18_ with 18 carbon lipophilic chain (PEG-stearate), PEG-C_16_ with 16 carbon lipophilic chain (PEG-palmitate) and PEG-OH without lipophilic chain [39]. They appeared in the chromatogram (Figure 1) as three peaks at 1.9, 6.4, and 7.2 min respectively. SA-PEG_100_-GalNAc appeared in the chromatogram as a single peak at 5.8 min. Suppocire^TM^ NB is composed of a mixture of di- and triglycerides of various acyl chain lengths (C_8_–C_18_). It appeared in the chromatogram as 15 peak clusters in the range of 8 to 26 min, and elution occurred according to the equivalent carbon number. Super refined soybean oil^TM^ was identified as being a mixture of C_16_–C_18_ unsaturated triglycerides, and appeared in the chromatogram as eight peak clusters in the range of 17 to 24 min [39]. Lipoid ^TM^ s75 is a phospholipid and is then mainly composed of phosphatidylcholine with a slight proportion of phosphatidylethanolamine and lysophosphatidylcholine. It appeared as a peak cluster between 8 min and 12 min. Lipoid ^TM^ s75 chromatogram was then overlapping Suppocire^TM^ NB peaks but it did not affect the quantification process of Myrj^TM^ S40 and SA-PEG_100_-GalNAc **6**, as lipids, including Lipoid ^TM^ s75, were removed during NLC sample preparation before UPLC-ELSD analysis (see below). 

Interestingly, Myrj^TM^ S40 and SA-PEG_100_-GalNAc **6** appeared in a different non-overlapping area of the chromatogram (respectively 1.9, 6.4, and 7.2 min for Myrj^TM^ S40 compounds, and 5.8 min for SA-PEG_100_-GalNAc **6**), and therefore, respective quantification was possible. To examine hypothetic interferences between components of disassembled NLC that would modify retention times, a mixture with 96/4 molar ratio of Myrj^TM^ S40 and SA-PEG_100_-GalNAc was analyzed by UPLC-ELSD (Supplementary Information, Figure S2). No interference among peaks was observed in the chromatogram. These results demonstrated the negligible influence of the matrix on the SA-PEG_100_-GalNAc **6** and Myrj^TM^ S40 quantification process. 

#### 2.3.2. Calibration Curves for Myrj^TM^ S40 and SA-PEG_100_-GalNAc 6

**Figure 2:**
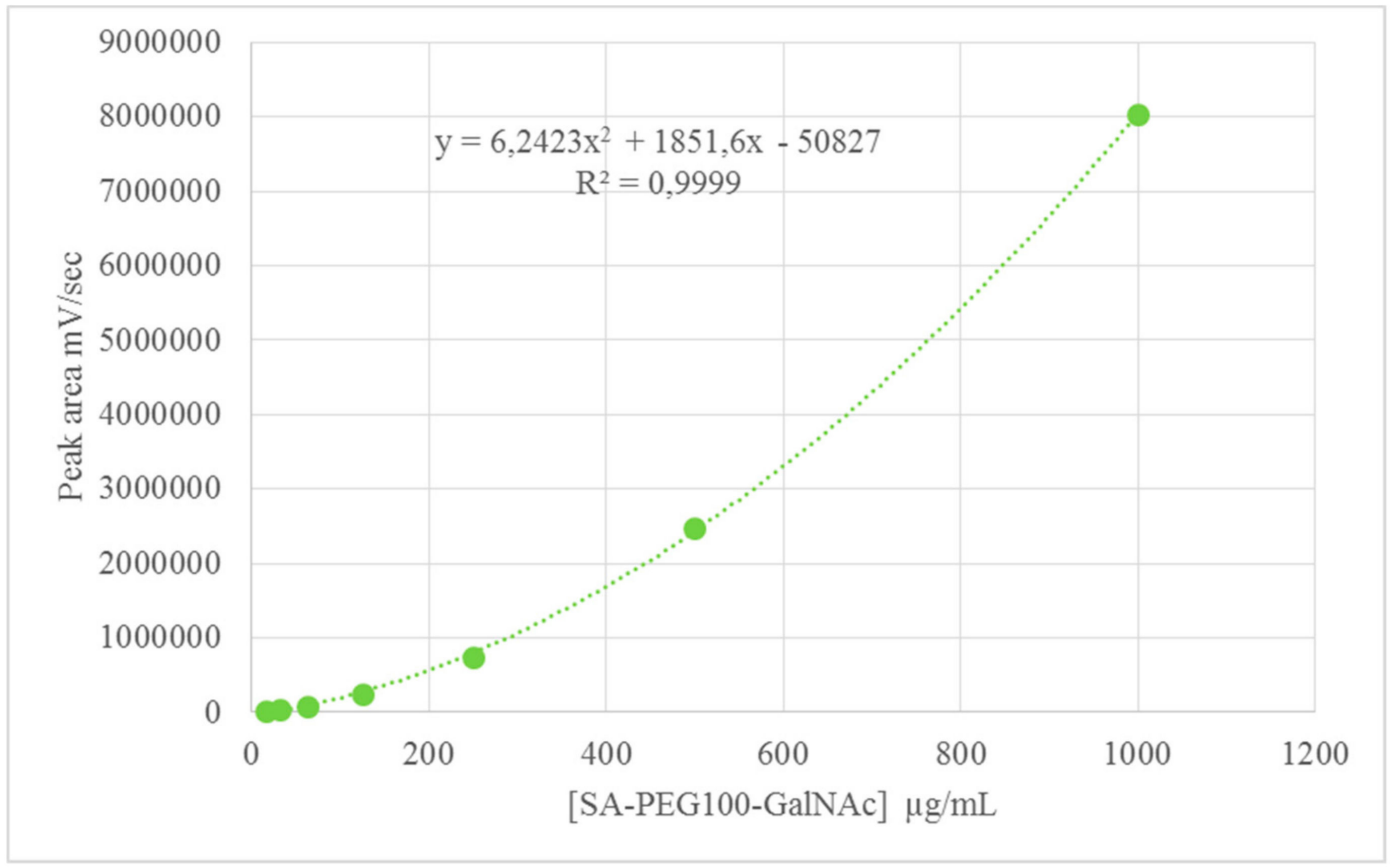
Calibration curve of free SA-PEG_100_-GalNAc **6**.

To quantify the amount of SA-PEG_100_-GalNAc **6** and the components of Myrj™S40 (PEG_40_-OH, PEG_40_-C_16,_ and PEG_40_-C_18_) at the NLC surface, calibration curves were established for these compounds by triplicating at seven concentrations from 15.6 µg/mL to 1 mg/mL obtained by half cascade dilution from stock solutions. Calibration curves for SA-PEG_100_-GalNAc **6**, Myrj™S40, and each its components (PEG_40_-OH, PEG_40_-C_16,_ and PEG_40_-C_18_) are reported respectively in Figure 2 (SA-PEG_100_-GalNAc **6**), and Figures S3 and S4 (Supplementary Information, Myrj™S40 components). The peak areas of SA-PEG_100_-GalNAc **6**, PEG_40_-OH, PEG_40_-C_16_ and PEG_40_-C_18_ were plotted against surfactant concentration. As previously used, a second-order polynomial (quadratic) was used as a fitting model [39], producing an excellent correlation coefficient (R^2^ values comprised between 0.9999 and 0.9994) (Figure 2, Figures S3 and S4). 

Equation of the calibration curves for SA-PEG_100_-GalNAc **6** and Myrj^TM^ S40 components were established (Figure 2, Figures S3 and S4). Additionally, limits of detection (LOD) were measured as 0.8 and 3.5 µg/mL, respectively for PEG-OH and SA-PEG_100_-GalNac **6**, and limits of quantification (LOQ) were 2.4 and 10.7 µg/mL respectively. Analyses were repeatable (relative standard deviation (RSD) of 1.4% and 1.5%, respectively for PEG-OH and SA-PEG_100_-GalNAc **6**). The values obtained for PEG-OH were in agreement with a previous study [39]. Once established and validated, the analytical method was used to quantify the exact percentage of SA-PEG_100_-GalNAc **6** and components of Myrj^TM^ S40 that were incorporated into NLC.

#### 2.3.3. Sample Preparation

**Figure 3:**
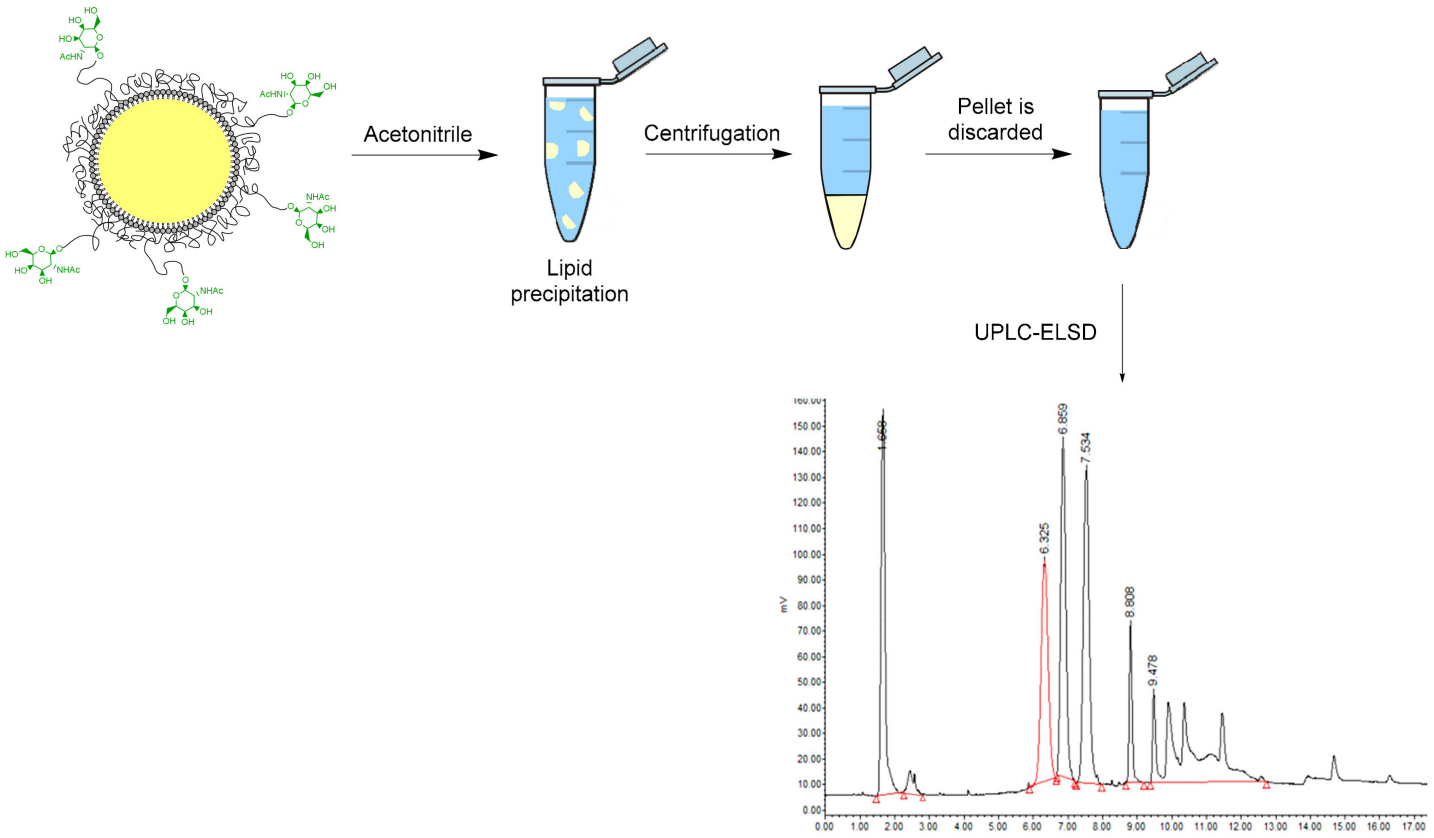
Sample preparation for the UPLC-ELSD analysis of SA-PEG100-GalNAc **6** and Myrj™S40 components in disassembled NLC (PEG Gal-NAc peak underlined in red in the chromatogram).

To analyze nanoparticle components, NLC were first disassembled by precipitation with acetonitrile. Supernatants containing SA-PEG_100_-GalNAc **6** and Myrj^TM^ S40 were analyzed by UPLC-ELSD after appropriate dilution (Figure 3). The pellets containing lipids (Lipoid ^TM^ s75, Suppocire^TM^ NB and Soybean oil^TM^) were analyzed to make sure that no SA-PEG_100_-GalNAc **6** or Myrj^TM^ S40 was precipitated in the pellet. Free SA-PEG_100_-GalNAc **6** and Myrj^TM^ S40 underwent the same treatment to assess the stability of these compounds during precipitation and no degradation was observed.

#### 2.3.4. Quantification Results

Chromatograms of disassembled NLC are reported in Supplementary Information Figure S5. The integration of peak areas corresponding to SA-PEG_100_-GalNAc **6** and Myrj^TM^ S40 components enabled the quantification of SA-PEG_100_-GalNAc **6** on the surface of the different formulations. For all analyzed formulations, obtained concentrations were above the LOQ, both for PEG-OH and SA-PEG_100_-GalNac **6**. The analyses of all formulations A–D were repeatable (RSD < 2%). Calculations are reported in Supplementary Information, Tables S1–S3.

**Figure 4:**
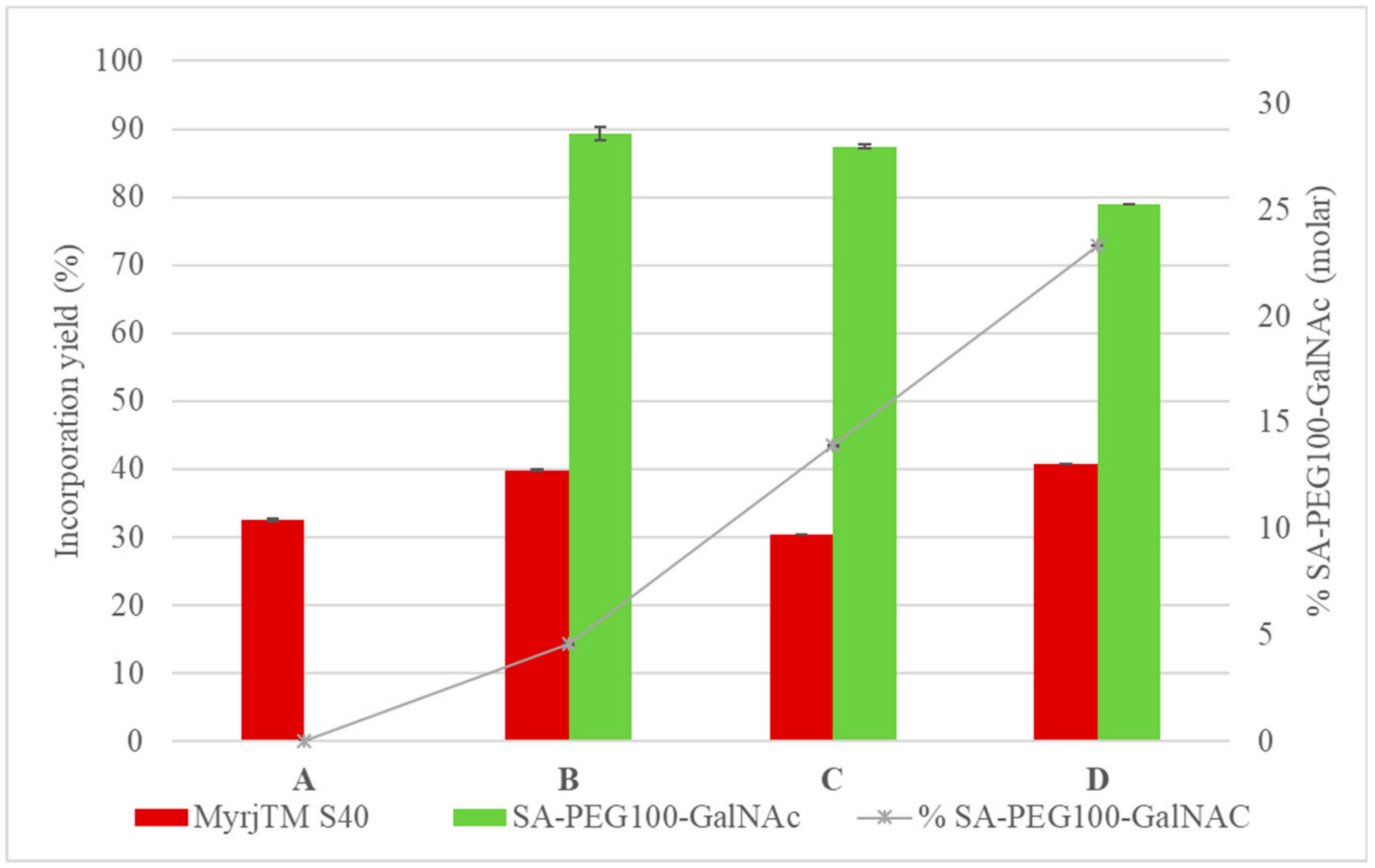
Quantification results on disassembled NLC (error bars: Standard deviation of three different experiments).

**Figure 5:**
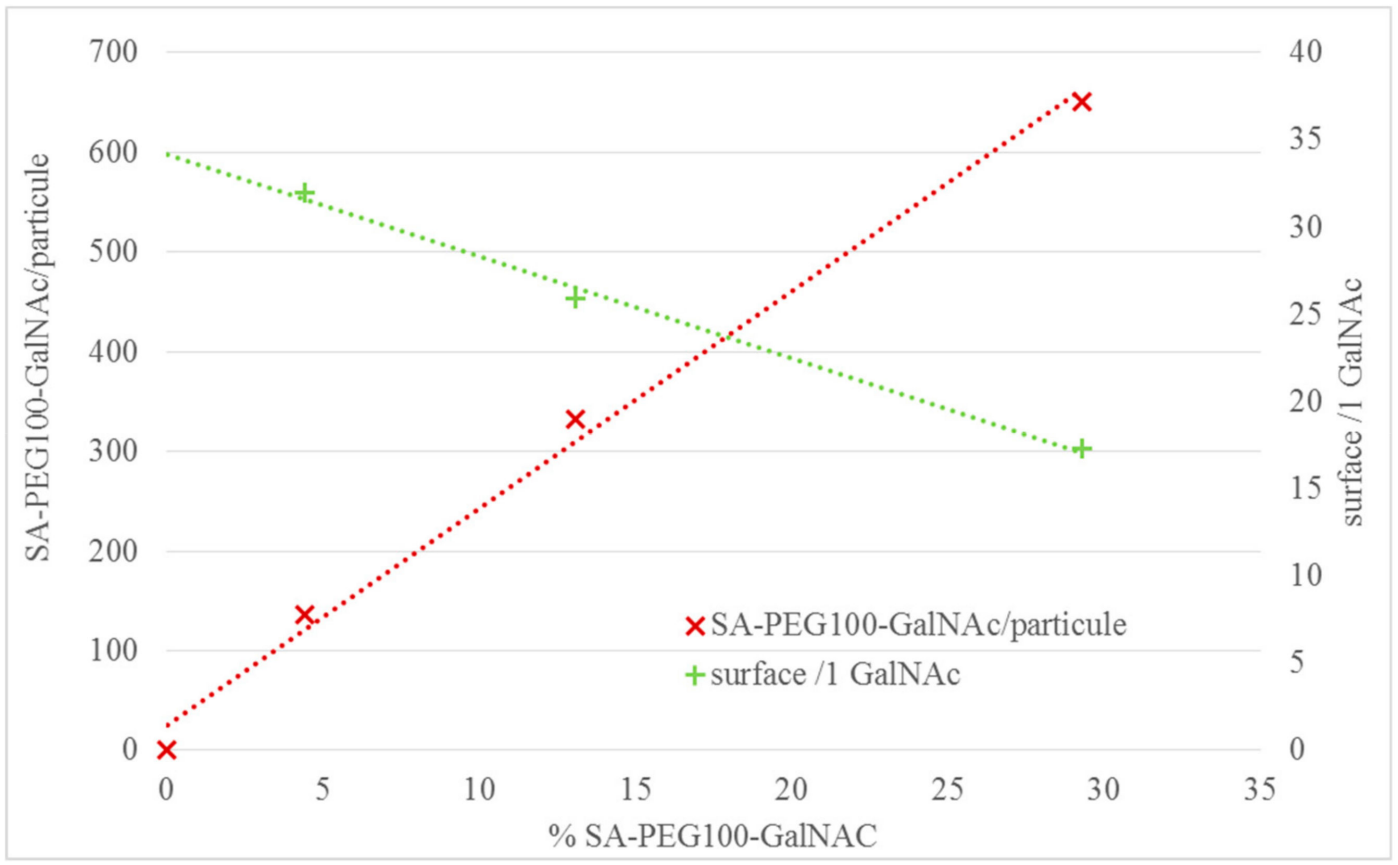
Surface density of SA-PEG_100_-GalNAc **6** per particle.

Incorporation yields and experimental molar % of SA-PEG_100_-GalNac **6** in the different formulations are plotted in Figure 4. Incorporation yields in formulations B, C, and D, showed similar results with about 80%–90% for SA-PEG_100_-GalNAc **6**, lower values from 32% to 41% were obtained for Myrj^TM^ S40 (Figure 4, Table S1). Formulations B, C, and D were initially formulated with 2.0%, 5.3% and 13.6 molar % of SA-PEG_100_-GalNAc **6** respectively. Because of the larger incorporation yield of SA-PEG_100_-GalNAc **6** compared to Myrj^TM^ S40, Formulation B finally exhibited 4.6% of SA-PEG_100_-GalNAc, Formulation C 13.9%, and Formulation D 23.3% (Supplementary Information, Table S1). In formulated nanoparticles, these values corresponded to 135 SA-PEG_100_-GalNAc **6** at NLC surface of Formulation B (1 GalNAc every 32 nm²), 333 SA-PEG_100_-GalNAc **6** for Formulation C (1 GalNAc every 26 nm²) and 650 SA-PEG_100_-GalNAc **6** for Formulation D (1 GalNAc every 17 nm²) (Supplementary Information, Tables S2 and S3, Figure 5).

## 3. Discussion

Nanostructured lipid carriers were designed, made of a lipid core composed of a mixture of liquid (soybean oil) and solid (Suppocire™ NB) lipids, stabilized by a layer of surfactants which contained GalNAc-modified ligands to actively target liver hepatocytes over-expressing ASGPR. Except for SA-PEG_100_-GalNAc **6** synthesized specifically for the study and whose toxicity is unknown, the commercial components used for NLC formulation are non-toxic, biocompatible, and FDA (Food and Drug Administration) or EMA (European Medicines Agency) approved. NLC present numerous advantages such as biocompatibility and in vivo tolerance [7,12], controlled drug release by tuning solid/liquid lipid ratio [40], and the possibility to be produced at middle to large scales by high-pressure homogenization [41]. 

Surface functionalization of lipid-based nanocarriers can be achieved by various non-covalent or covalent surface engineering techniques [18]. One is the covalent attachment of targeting ligands following the synthesis of nanocarriers presenting functional chemical groups on their surface. This covalent ligation is usually based on classical “click reactions” that can be performed in water where the nanocarriers are suspended. Additionally, such mild conditions are also favorable as they do not damage the structure of fragile targeting moieties, such as proteins. Classical reactions include amide bond formation, thiol-maleimide Michael addition, disulfide bond formation, hydrazine and oxime ligation, azide-alkyne 1,3 dipolar cycloaddition [42,43]. For example, amino-functionalized preformed solid lipid nanoparticles have been previously decorated by Gal moieties taking benefit of the D-galactose open-chain form comprising aldehyde group, involving the formation of a Schiff’s base, further reduced to a secondary amine [34]. 

The “post-synthesis functionalization” strategy is the preferred route for the attachment of targeting ligands on inorganic nanocarriers or some polymer nanoparticles. However, the “pre-functionalization” strategy is generally preferred for liposomes or solid lipid nanoparticles, unless the targeting moiety to attach is a very precious biological material, introduced in very low quantity at the nanocarrier surface, or can be degraded during the nanoparticle assembly process. Indeed, in the latter strategy, the characterization of ligand-modified surfactants can be carefully performed by usual techniques such as NMR, and the quantity of targeting ligands introduced during the formulation well controlled. Using this strategy, SiRNA/lipid nanoparticles decorated by PEGylated-GalNAc surfactants were designed to deliver genes in hepatocytes [44,45]. Gal targeting moieties were also introduced at the surface of different liposomes or lipid nanoparticles using Gal derivatives of cholesterol [38], alpha-tocopherol [46], or DSPE (distearoyl phosphatidylethanolamine) [35,36,47]. However, the number of Gal or GalNAc moieties introduced at the surface of the nanoparticles was not experimentally quantified, and when calculated, total incorporation of surfactants in the formulation was assumed [35]. 

In the present study, the “pre-functionalization strategy” was used. GalNAc-functionalized PEGylated surfactants were designed, synthesized, and characterized, before being introduced during the formulation of nanostructured lipid carriers in different proportions. GalNAc was used as a decorating ligand since it displays about 50-fold higher affinity for ASGPR than Gal [22,23,24]. Attachment of the GalNAc moiety to the PEG chain was performed in acetonitrile/water mixture by the classic thiol-maleimide coupling. Experiments using dibenzocyclooctyne (DBCO) terminated surfactant for copper-free click ligation were performed as well but stopped rapidly due to the poor stability of the DBCO derivative in aqueous media. The PEG-GalNAc surfactants were designed with longer PEG chains (about 100 ethylene oxide units) than this stabilizing nanoparticle surface (Myrj™S40, around 40 ethylene oxide units), to promote efficient interactions of GalNAc moieties with ASGPR expressed on the cell surface. However, when increasing the percentage of long PEG surfactants, nanoparticle diameter increased whereas their colloidal stability decreased (Table 2), as previously observed [11]. This could be accounted for by a change of oil/water interface tension and/or PEG conformation (from coil to brush for longer chains). An optimal functionalization ratio was therefore defined at 10% molar of SA-PEG_100_-GalNAc **6** introduced during formulation.

To get a precise characterization of the nanoparticles and measure the quantity of GalNAc moieties actually introduced on the NLC surface, namely the ligand density, a UPLC-ELSD quantitative method was developed. It allowed to demonstrate that SA-PEG_100_-GalNAc **6** was incorporated at 80%–90% in formulations B, C, and D. Myrj™S40 had a lower incorporation yield from 32% to 41% in formulations A, B, C, and D. This lower percentage can be partly explained by the composition of the commercial Myrj™ S40 product. As recently reported [39], this component is composed of three different compounds: PEG_40_-OH, PEG_40_-C_16,_ and PEG_40_-C_18_ in approximately equal quantities (30.1% w/w, 34.6% w/w, and 35.3% w/w, respectively). Thanks to their aliphatic C_16_ or C_18_ chain that interacts with Lipoid ^TM^ s75 phospholipid chain and triglycerides, PEG-C_16_ and PEG-C_18_ are quite well anchored at the lipid core/water interface of the nanodroplets, similarly to SA-PEG_100_-GalNAc **6**. On the other hand, PEG-OH, because of its lack of lipophilic anchor, is eliminated during purification of the formulation by dialysis [39]. Therefore, at the end of the purification step, SA-PEG_100_-GalNAc **6**/Myrj™ S40 molar ratio was increased in comparison to ratios initially introduced for the formulation step (4.6% versus 2.1% for Formulation B, 13.9% versus 5.3% for Formulation C, and 23.3% versus 13.6% for Formulation D). From the above quantification data of the surfactants and particle diameter determined by DLS, the number of GalNAc moieties introduced and their density on the NLC surface can be calculated (Supplementary Information, Tables S2 and S3). The particle hydrodynamic diameter increased significantly when the number of GalNAC units increased per particle (from 40 to 60 nm), and this effect was taken into account for the GalNAc surface density calculation. Formulations of nanoparticles exhibited then 1 GalNAc every 32 nm² (Formulation B), 1 GalNAc every 26 nm² (Formulation C), and 1 GalNAc every 17 nm² (Formulation D). The mean distance between GalNAc units could be estimated to be 5.6 nm (Formulation B), 5.2 nm (Formulation C), and 4.1 nm (Formulation D).

Multivalent interactions with the ASGPR, and therefore the targeting efficiency, are highly dependent on the number and the spatial arrangement of the monosaccharide ligands [26,27,28,29,30,31,32,33]. The “optimal distance” between Gal units to benefit from multivalent interactions with the receptor was estimated by Lee et al. to be 15–20 Å apart in a triangle geometry [30,31]. 

Several GalNAc- or Gal-functionalized lipid nanoparticles described in the literature have already demonstrated their interest in delivering therapeutic compounds to the liver, and their ASGPR recognition efficiency was assumed to be correlated with the surface density of Gal or GalNAc residues [29]. However, no quantification protocol was developed, and only in a few cases, the number of Gal or GalNAc residues were roughly estimated based on the hypothesis of full incorporation of saccharide-surfactants at the nanoparticle surface [35,36,38,44,45,46,47]. Among these studies, the Gal ligand density decorating the surface of lipid nanocapsules investigated by Morille et al. for gene therapy can be calculated from reported data, assuming total incorporation of the saccharide moieties (Supplementary Information, Table S4, Figure S6) [35]. Interestingly, these data described nanoparticles with lower density (at best 1 Gal ligand/57 nm^2^) than herein quantified at the NLC surface (at least 1 GalNAc ligand/32 nm^2^). Our system, therefore, improves the GalNAc-GalNAc distance (about 4 nm), and is closer to the “optimal distance” estimated by Lee et al. (15–20 Å) [30,31] to benefit from multivalent interactions. Morille et al. also reported that DSPE-PEG_2000_-Gal coating at 1 ligand/57 nm^2^ did not induce significant effect in improving lipid nanocapsule transfection efficiency in primary rat hepatocytes, whereas F108-Gal coated nanocapsules (1 ligand/107 nm^2^) strongly improved gene delivery These results, not correlated to the Gal ligand surface density, suggested a difference in sugar accessibility. They illustrate the importance of the spatial arrangement of the terminal Gal units and highlight the complexity of these systems and their comparison. For herein described NLC, the use of a surfactant-containing about 100 ethylene oxide units should make the GalNac ligands more accessible to the receptor. In addition, in comparison to previously described nanoparticles [35,36,38,44,45,46,47], their smaller size, their higher surface density and the use of GalNac having a 50 times higher affinity than Gal, should lead to improved multivalent interaction with the ASGPR. Future studies will be directed to evaluate the ability of these GalNAc-decorated NLC to efficiently enter hepatic cells. 

## 4. Materials and Methods 

### 4.1. General Information

Chemical reactants and solvents were purchased from Sigma-Aldrich (Saint Quentin Fallavier, France) and were used without further purification unless specified. Compound **5** was obtained according to previously reported procedures [33]. BocNH-PEG_100_-NH_2_ was purchased from Iris Biotech GmbH (Marktredwitz, Germany). Suppocire^TM^ NB and Labrafac were purchased from Gattefossé (Saint-Priest, France). Myrj^TM^ S40 and Myrj^TM^ S100 (poly(ethylene glycol) surfactants with respectively about 40 and 100 ethylene oxide units), and super-refined soybean oil were supplied by CRODA (Chocques, France). Lipoid^TM^ s75 was purchased from Lipoid GmbH (Ludwigshafen, Germany). SpectraPor^TM^ dialysis membrane of 12–14,000 MWCO was purchased from Roth Sochiel EURL (Lauterbourg, France). HPLC grade solvents were obtained from VWR Scientific (Fontenay-sous-Bois, France).

All water solutions were prepared from ultra-pure laboratory-grade water that has been filtered and purified by reverse osmosis using Millipore Milli-Q^TM^ cartridge system (resistivity 18.2 MΩ.cm at 25 °C, Merck, Fontenay-sous-bois, France). Thin-layer chromatography (TLC) was performed on silica gel 60 F254 (Merck). ^1^H NMR spectra were recorded on a Bruker Avance 400 spectrometer (Bruker, Wissembourg, France). Chemical shifts (δ) are reported in ppm with the solvent as the internal reference. 

### 4.2. Synthesis

#### 4.2.1. SA-PEG_100_-NHBoc 2

To a solution of stearic acid (1.03 g, 3.60 mmol) in CH_2_Cl_2_ (80 mL), benzotriazol-1-yloxy)tris(dimethylamino)phosphonium hexafluorophosphate (BOP, 1.60 g, 3.60 mmol) was added. After complete dissolution, commercially available BocNH-PEG_100_-NH_2_ **1** (5.0 g, 1.03 mmol) and N,N-diisopropylethylamine (DIEA; 470 mg, 3.60 mmol) were added. After stirring for 2 h at room temperature, the resulting mixture was concentrated under reduced pressure. Precipitation was obtained upon the addition of cold diethyl ether. The precipitate was filtered, dissolved in water and purified by dialysis for 48 h versus a large volume of water (MW cut-off 1 kDa). After lyophilization, SA-PEG_100_-NHBoc **2** was obtained as a white powder (3.15 g, 0.63 mmol, 61% yield). 

^1^H NMR (300 MHz; CDCl_3_)—δ: 0.87 (t, J = 7.2 Hz; 3H), 1.13–1.36 (m, 28H), 1.44 (s, 9H), 1.60 (quint, J = 15.1, 7.6 Hz, 2H), 2.42 (t, J = 7.5 Hz, 2H), 3.30 (t, J = 5.0 Hz, 2H), 3.40 (t, J = 5.0 Hz, 2H), 3.48–3.80 (m, 360H), 3.87 (t, J = 5.0 Hz, 2H), 7.79 (bs, 1H).

#### 4.2.2. SA-PEG_100_-NH_3_^+^TFA^−^ 3

To a solution of SA-PEG_100_-NHBoc **2** (1.61 g, 0.30 mmol) in CH_2_Cl_2_ (10 mL), trifluoroacetic acid (TFA, 6.32 g, 55 mmol) was added. After stirring for 1 h at room temperature, the resulting mixture was concentrated under reduced pressure, and precipitation was obtained upon addition of cold diethyl ether. The precipitate was filtered, dissolved in water, and purified by dialysis for 48 h versus a large volume of water (MW cut-off 1 kDa). After lyophilization, SA-PEG_100_-NH_3_^+^TFA^-^ **3** was obtained as a white powder (1.37 g, 0.26 mmol, 85% yield). 

^1^H NMR (400 MHz; CDCl_3_)—δ: 0.87 (t, J = 7.2 Hz, 3H), 1.13–1.36 (m, 28H), 1.60 (quint, J = 15.1, 7.6 Hz, 2H), 2.15 (t, J = 7.5 Hz, 2H), 3.17 (bt, 2H), 3.4 (m, 4H), 3.48–3.80 (m, 360H), 3.87 (t, J = 5.0 Hz, 2H), 6.14 (bs, 1H), 7.90 (bs, 2H).

#### 4.2.3. SA-PEG_100_-Maleimide 4

To a solution of 6-maleimidohexanoic acid (8.40 mg, 0.040 mmol) in CH_2_Cl_2_ (5 mL), BOP (17.5 mg, 0.04 mmol) was added After stirring for 10 min under argon atmosphere, SA-PEG_100_-NH_3_^+^TFA^-^ **3** (100 mg, 0.020 mmol) and DIEA (5 µL, 0.040 mmol) were added. The reaction mixture was stirred for 1 h until disappearance of starting SA-PEG_100_-NH_3_^+^TFA^-^ **3** (TLC SiO_2_, eluent CH_2_Cl_2_/CH_3_OH 9/1 v/v, ninhydrine). The resulting mixture was concentrated under reduced pressure and precipitation was obtained upon the addition of cold diethyl ether. SA-PEG_100_-maleimide **4** was obtained as a white powder (97.6 mg, 0.02 mmol, 94% yield).

^1^H NMR (400 MHz, CDCl_3_)—δ: 0.87 (t, J = 6.7 Hz, 3H), 1.13–1.36 (m, 28H), 1.62 (qd, J = 15.1, 7.6 Hz, 6H), 1.80 (t, J = 6.4 Hz, 2H), 2.15 (t, J = 7.6 Hz, 4H), 3.04–3.21 (m, 4H), 3.40–3.48 (m, 5H), 3.50–3.75 (m, 360H), 3.81 (t, J = 5.6 Hz 3H), 6.14 (s, 1H), 6.68 (s, 2H).

#### 4.2.4. SA-PEG_100_-GalNAc 6

To a solution of SA-PEG_100_-maleimide **4** (97.6 mg, 0.019 mmol) and TCEP (23.5 mg, 0.094 mmol) in CH_3_CN (3 mL), a solution of GalNAc-SSPy derivative **5** (18 mg, 0.038 mmol) in H_2_O (1 mL) was added dropwise under argon atmosphere. The resulting yellow solution was stirred at room temperature for 5 h. The mixture was concentrated under reduced pressure, and precipitation was obtained upon the addition of cold diethyl ether. The precipitate was filtered, dissolved in water, and purified by dialysis for 48 h versus a large volume of water (MW cut-off 1 kDa). SA-PEG_100_-GalNAc **6** was obtained as a white powder (87.2 mg, 0.016 mmol, 87% yield). 

^1^H NMR (400 MHz, D_2_O) δ: 0.82 (t, J = 6.4 Hz, 3H), 1.21 (s, 28H), 1.42–1.61 (m, 5H), 2.08–2.20 (m, 3H), 2.38 (dd, J = 17.4, 9.3 Hz, 1H), 2.43–2.55 (m, 4H), 2.56–2.68 (m, 4H), 3.12 (d, J = 12.7 Hz, 1H), 3.19 (d, J = 6.7 Hz, 1H), 3.30 (t, J = 5.4 Hz, 3H), 3.41–3.47 (m, 3H), 3.50 (t, J = 5.5 Hz, 2H), 3.52–3.56 (m, 2H), 3.62 (s, 360H), 3.80 (dd, J = 5.4, 3.5 Hz, 3H), 4.42 (d, J = 4.5 Hz, 1H).

### 4.3. Formulation of Nanostructured Lipid Carriers

The lipid phase was prepared by mixing solid (Suppocire™ NB) and liquid (super-refined soybean oil) glycerides as well as the lipophilic surfactant Lipoid™ s75, while the aqueous phase was composed of the hydrophilic surfactant, Myrj^TM^ S40, eventually SA-PEG_100_-GalNAc **6** or Myrj^TM^ S100 and 1X PBS aqueous buffer (100 mM phosphate, NaCl 154 mM, pH 7.4). After homogenization at 45 °C, both lipid and aqueous phases were crudely mixed and sonication cycles were performed at 45 °C during 5 min. A conical tip sonicator instrument (AV505 ultrasonic processor, Sonics, Newton, Connecticut) with a 3 mm diameter probe was used. Non encapsulated components were separated from LNP by dialysis (1× PBS, MW cut-off: 12,000–14,000 Da, overnight). Prior to characterization, LNP dispersions were diluted to obtain 5 mL and were then filtered through a 0.22 µm cellulose Millipore membrane. Particle concentration was assessed by weighting freeze-dried samples of NLC obtained from a known volume.

### 4.4. Dynamic Light Scattering

The hydrodynamic diameter and zeta potential of NLC were measured at 22 °C with a Malvern Zeta Sizer Nano instrument (NanoZS, Malvern, Orsay, France) in 0.1× PBS buffer. Physical stability was investigated by DLS measurements over one year with samples stored at 4 °C. At least three different NLC batches (2 mL, lipid dispersed phase weight fraction: 10%) were used per condition. Mean average diameters and polydispersity indices reported were obtained from scattered light intensity results. Data were expressed in terms of mean and standard deviation of all the samples for each condition, each sample result being the mean of three independent measurements performed at 25 °C.

### 4.5. UPLC-ELSD Analysis

#### 4.5.1. Sample Preparation

Each of the standards (Myrj^TM^ S40, SA-PEG_100_-GalNAc **6**, Suppocire™ NB, super-refined soybean oil and Lipoid s75™) was weighed on a calibrated, analytical balance and dissolved in a mixture of CHCl_3_/MeOH 2/1 (*v*/*v*) to give 1 mg/mL stock solutions. For UPLC-ELSD analysis, nanoparticles were disassembled. Three hundred µL of nanostructured lipid carriers were added to 1700 µL of acetonitrile. Samples were centrifuged to pellet the lipids after precipitation. Five hundred µL of supernatant was added to 1500 µL acetonitrile, and the samples were centrifuged once more to remove any remaining lipid in the supernatant. Two hundred fifty µL of supernatant was concentrated under reduced pressure and dissolved in 500 µL of a mixture of CHCl_3_/MeOH 2/1 (*v*/*v*) to yield solutions with approximatively 1 mg/mL concentration of total PEGylated surfactants (Myrj^TM^ S40 and **6**) before UPLC-ELSD analysis.

#### 4.5.2. Chromatographic Conditions

The chromatographic analysis of the NLC ingredients was performed using an Acquity® UPLC HClass system (Waters, Guyancourt, France) coupled with an Alltech 3300 evaporating light scattering detector (ELSD, Grace, Buchi, Rungis, France). Separation of the different components was achieved using a CORTECS UPLC-C18 column (1.6 µm, 150 × 2.1 mm, 90 Å, Waters). Eluents were solvent A: Deionized water, B: Methanol, and C: Mixture of isopropanol/acetonitrile 75/25 (*v*/*v*). Gradients are displayed in Table 3. The drift tube was set at 45 °C with a flow of N_2_ set at 2.0 L/min and a gain at 4. The injected volume was 5 µL and the column temperature was set at 40 °C. 

#### 4.5.3. Calibration Curves

Stock solutions of SA-PEG_100_-GalNAc **6** and Myrj^TM^ S40 (1000 μg/mL in CHCl_3_/MeOH 2/1 (*v*/*v*)) were diluted in a CHCl_3_/MeOH mixture (2/1 *v*/*v*) to prepare standards. The calibration curves were prepared on the same day over the range of 15.625–1000 μg/mL. For validation, each concentration point was run in triplicate. Average peak area versus sample concentration curves was plotted to establish calibration curves.

#### 4.5.4. Validation of Analytical Method

The limit of detection was determined as previously described [39]. Since the whole range calibration curves were well fitted using only quadratic equations, only lower concentration calibration points (i.e., concentration range of 4–38 µg/mL and 15–63 µg/mL, respectively for PEG-OH and SA-PEG_100_-GalNac **6**) were used, for which linear calibration curves could be obtained with R^2^ > 0.99. The limits of detection (LOD) and quantification (LOQ) were thus estimated based on the standard deviations of the y-intercepts of regressions analysis (σ) and the slope (S), by the following equations LOD = 3.3 σ/S and LOQ = 10 σ/S. The repeatability (RSD) of the method was evaluated by three independent measurements of standard solutions (at 20 µg/mL and 62.5 µg/mL for PEG-OH and SA-PEG_100_-GalNac **6**, respectively) and NLC samples. Precision was expressed as the relative standard deviation (RSD, %) of the three independent measurements.

## 5. Conclusions

To our knowledge, it is the first time a full analytical method based on UPLC-ELSD was successfully used to quantify the precise amount of GalNAc units present on the surface of lipid nanoparticles. The obtained GalNAc-modified NLC of 50 nm diameter presented a quantifiable density of ligands comprising between 135 and 650 GalNAc per particle. Compared to reported studies, these data would suggest that these nanocarriers are promising vectors to target the ASGPR (asialoglycoprotein receptors) mainly expressed at the surface of hepatocytes. Further explorations of these nanocarriers, namely their ability to encapsulate and deliver drugs to treat liver-related pathologies, such as hepatocellular carcinoma, hemochromatosis, or Wilson’s disease, are currently under progress.

## Figures and Tables

**Table 1:** Composition of formulations A, B, C, and D (quantities in mg).

	Ingredients	A	B	C	D	E
	Soybean oil	85	85	85	85	85
Lipid phase	Suppocire™NB	245	245	245	245	245
	Lipoid™ s75	65	65	65	65	65
Aqueous phase	Myrj™S40	345	327	298.3	241.1	241.1
	SA-PEG_100_-GalNAc	0	18.7	46.7	105.4	-
	*SA-PEG_100_-GalNAc (% mol)*	*0*	*2.1*	*5.3*	*13.6*	*-*
	Myrj™S100	-	-	-	-	105.4
	PBS	qsp 2 mL	qsp 2 mL	qsp 2 mL	qsp 2 mL	qsp 2 mL

**Table 2:** DLS characterization of formulations A, B, C, and D.

	A	B	C	D	E
Hydrodynamic diameter (nm)	41.8 ± 1.3	37.2 ± 1.3	52.4 ± 1.4	59.8 ± 0.6	63.9 ± 1.5
PDI	0.11 ± 0.02	0.11 ± 0.03	0.11 ± 0.02	0.14 ± 0.02	0.18 ± 0.02
Zeta potential (mV)	−3.9 ± 1.3	−5.9 ± 2.1	−6.2 ± 2.5	−6.3 ± 2.4	−4.4 ± 2.5
Colloidal stability during storage at 4 °C	1 year	5 months	3 months	3 weeks	1 month

**Table 3:** UPLC gradient elution program for the analysis of NLC ingredients.

Time (min)	Flow (mL/min)	A	B	C
0	0.25	30	70	0
3	0.3	10	90	0
15	0.3	0	100	0
22	0.3	0	35	65
25	0.3	0	35	65
25.1	0.25	30	70	0
30	0.25	30	70	0

## Supplementary Materials

Supplementary materials can be found at https://www.mdpi.com/1422-0067/20/22/5669/s1.
