# Supplementary material for: Quantification of Surface GalNAc Ligands Decorating Nanostructured Lipid Carriers by UPLC-ELSD
Source: Int J Mol Sci. 2019 Nov 12;20(22):5669. doi: 10.3390/ijms20225669 (PMC6888163; doi:10.3390/ijms20225669)
Supplement: Supplementary file 1 [file ijms-20-05669-s001.pdf]

Quantification of surface GalNAc ligands decorating Nanostructured Lipid Carriers by UPLC-ELSD: Supplementary Information

Laura Gauthier[1,2], Mathieu Varache[1], Anne-Claude Couffin[1], Colette Lebrun[2], Pascale Delangle[2], Christelle Gateau[2*], Isabelle Texier[1*]

[1] Univ. Grenoble Alpes, CEA, LETI-DTBS, F-38000 Grenoble, France ; isabelle.texier-nogues@cea.fr
[2] Univ. Grenoble Alpes, CEA, CNRS, IRIG-SyMMES, F-38000 Grenoble, France ; christelle.gateau@cea.fr
* Correspondence: christelle.gateau@cea.fr, isabelle.texier-nogues@cea.fr ; Tel.: +33-438-786-041 (C.G.) ; +33-438-784-670 (I.T.)

NMR spectra of SA-PEG100-maleimide 4 and SA-PEG100-GalNAc 6

Figure S1 displays the NMR spectra of SA-PEG100-maleimide **4** (Figure S1 (a)) and SA-PEG100-GalNAc **6** (Figure S1 (b)). The disappearance of maleimide protons at δ ~ 6.7 ppm evidenced the grafting of the GalNAc moiety.

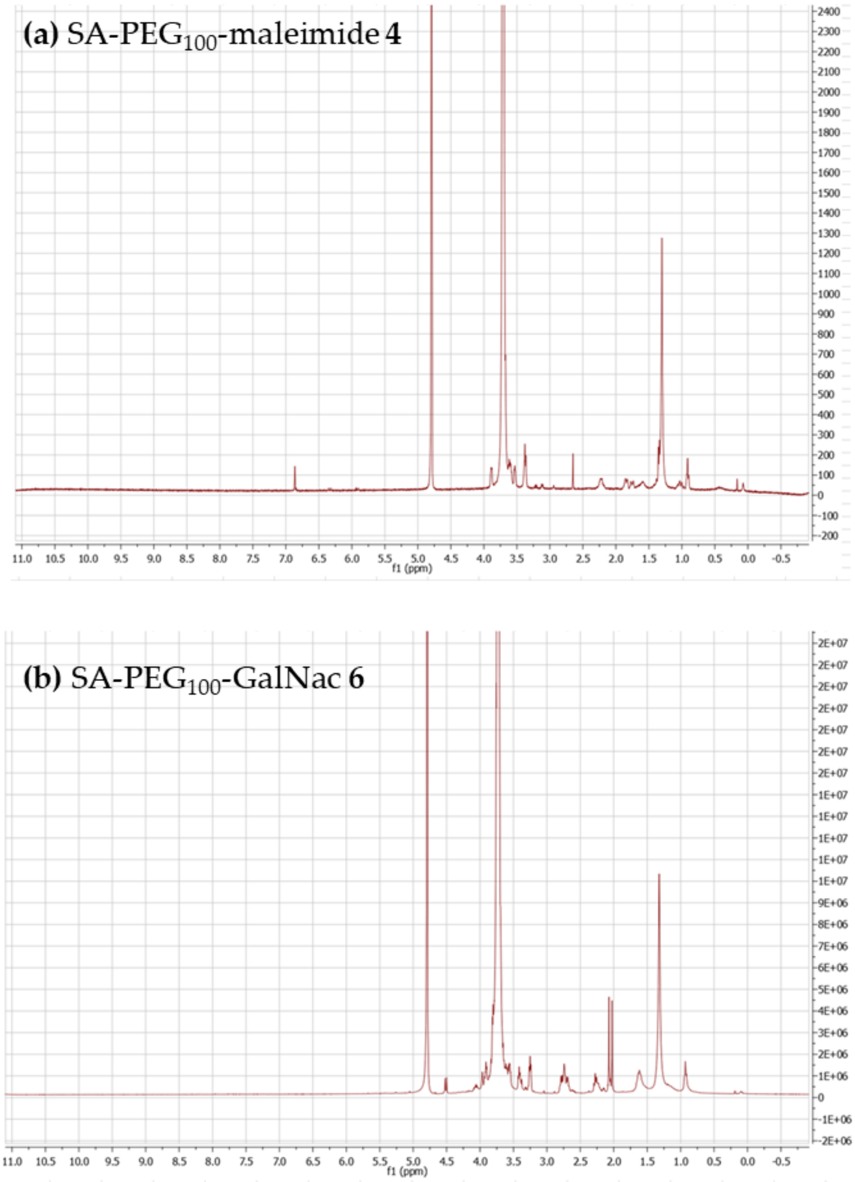

Figure S1. NMR spectra of SA-PEG100-maleimide **4** **(a)** and SA-PEG100- GalNAc **6** **(b).**

Analysis of mixture of Myrj™ S40 and SA-PEG100-GalNAc

The chromatogram of a 96/4 molar mixture of Myrj™ S40 and SA-PEG100-GalNAc is the sum of the chromatograms of the 2 separated products (Figure S2), evidencing no interference in their analysis when in mixture.

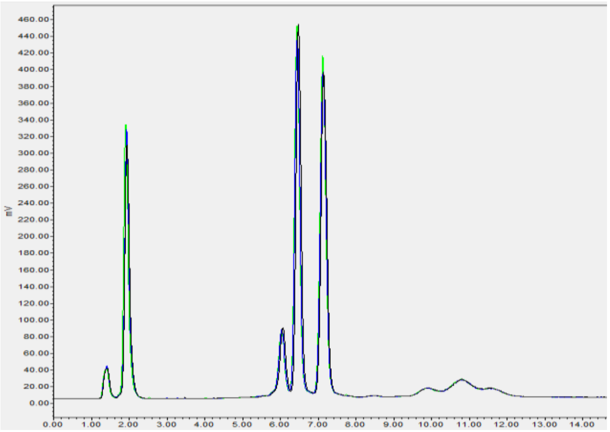

Figure S2. Chromatogram of a 96/4 molar mixture of Myrj™ S40 and SA-PEG100-GalNAc (total concentration: 1 mg/mL).

Additional UPLC-ELSD calibration curves

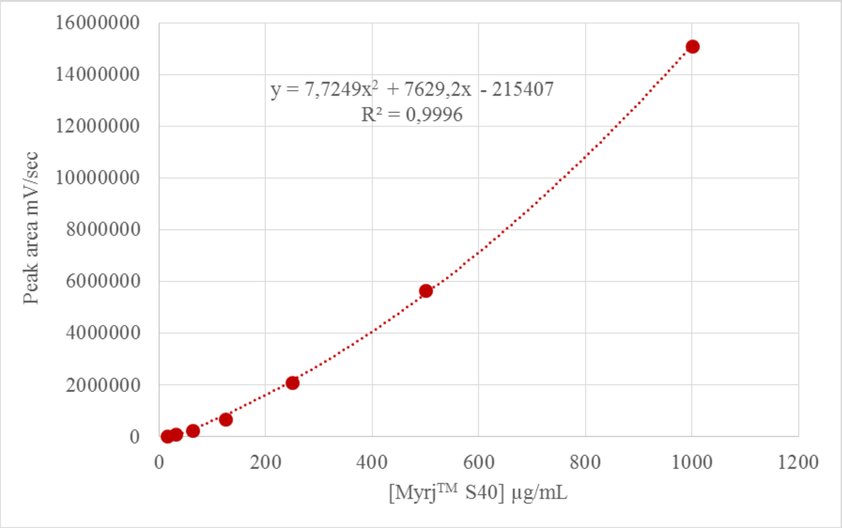

Figure S3. Calibration curve obtained for Myrj™ S40, when the 3 peaks of PEG-OH, PEG-C_{16}, PEG-C_{18} were summed.

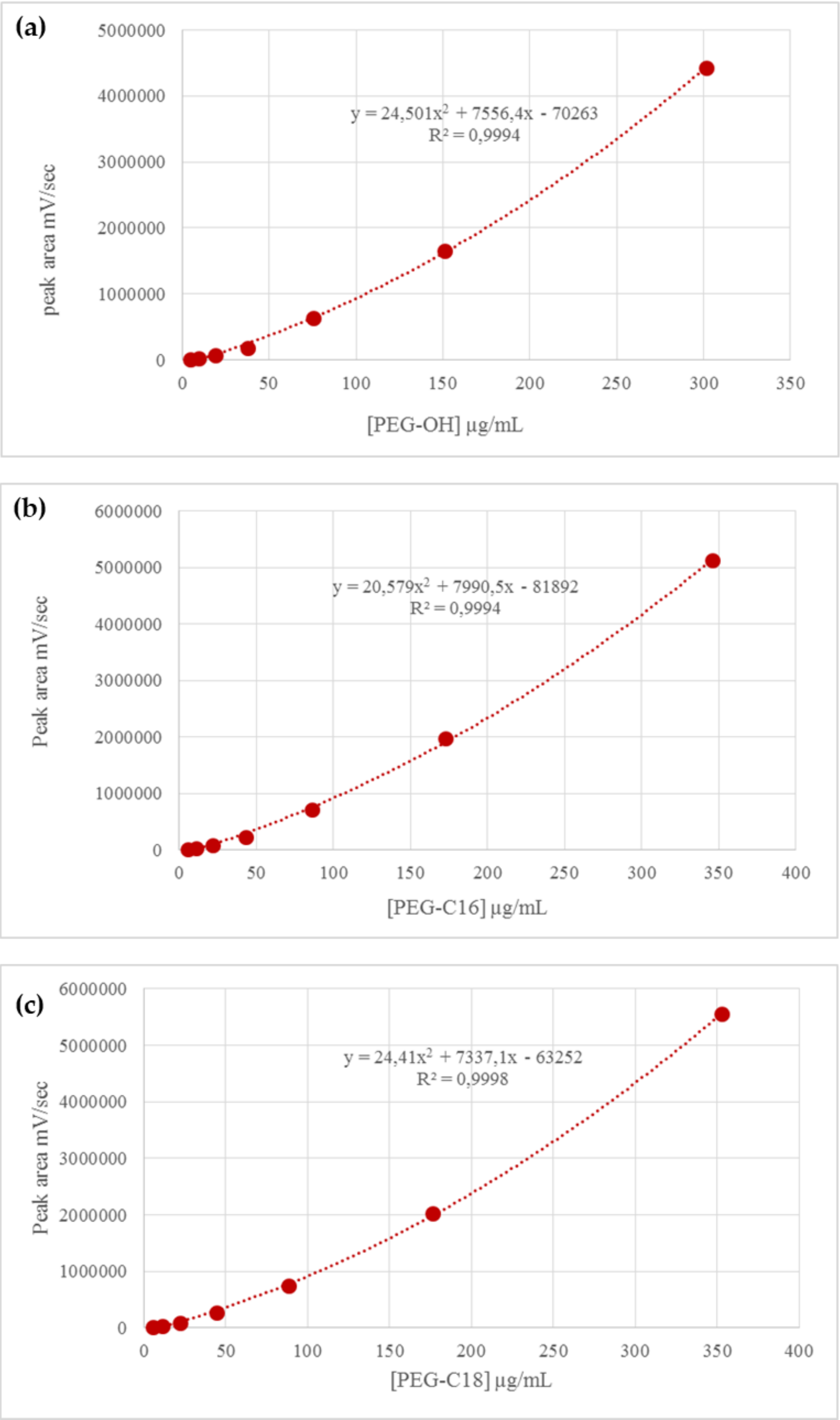

Figure S4. Calibration curves for each component of Myr™ S40: (a) PEG-OH, (b) PEG-C_{16}, (c) PEG-C_{18}.

UPLC-ELSD analysis of disassembled NLC

Figure S5 displays the chromatograms of disassembled NLC.

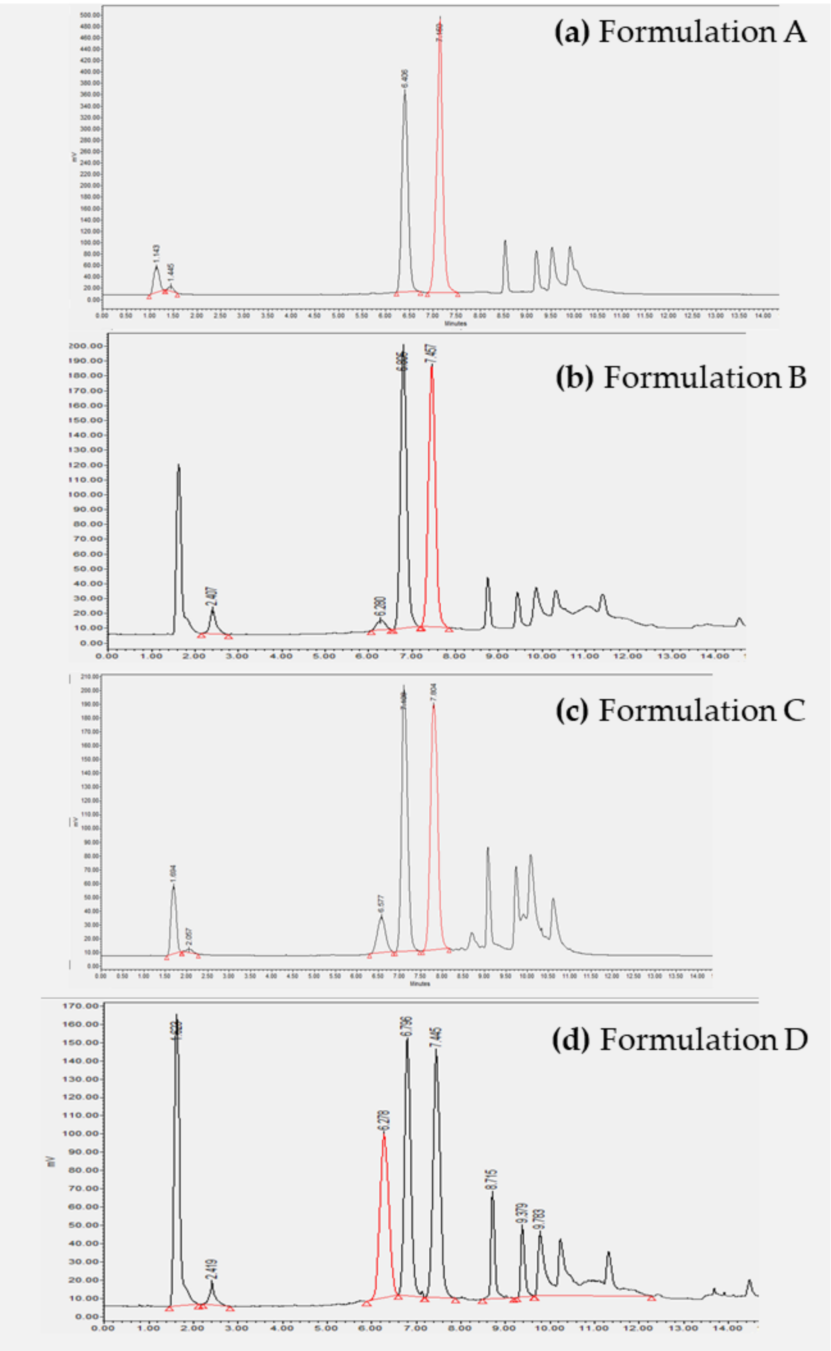

Figure S5. Chromatograms of disassembled NLC.

Table S1 summarizes the UPLC-ELSD quantification data for each formulation and details the calculation of the molar percentage of incorporated Myrj™ s40 and SA-PEG$_{100}$-GalNac.

Table S1. Quantification data for each disassembled formulation (mean ± standard deviation of three independent measurements).

		A	B	C	D
EXPERIMENTAL UPLC peak area	PEG-OH	209640 ± 877	197549 ± 2476	26023 ± 1715	83428 ± 7267
	PEG-C16	2025400 ± 16874	2413922 ± 13587	1147209 ± 57649	1570128 ± 11466
	PEG-C18	1993916 ± 26553	2732216 ± 1162	2113177 ± 23644	1921453 ± 3463
	Myrj™ S40	4228956 ± 31083	5343687 ± 12261	3286409 ± 80745	3575009 ± 21539
	SA-PEG$_{100}$ GalNAc	0	96180 ± 1023	378972 ± 22815	1133502 ± 8709
EXPERIMENTAL injected concentration (calculated with UPLC calibration curve) (µg/mL)	PEG-OH	33.42 ± 0.14	32.04 ± 0.40	12.26 ± 0.04	19.15 ± 0.09
	PEG-C16	180.15 ± 1.50	203.26 ± 1.14	117.98 ± 0.32	149.32 ± 0.06
	PEG-C18	176.61 ± 2.35	217.55 ± 0.09	184.00 ± 0.11	172.04 ± 0.02
	Myrj™ S40	390.18 ± 2.87	452.85 ± 1.04	314.23 ± 0.41	340.51 ± 0.11
	SA-PEG$_{100}$ GalNAc	0.00	65.10 ± 0.69	153.10 ± 0.49	311.82 ± 0.13
EXPERIMENTAL concentration in NLC sample (mg/mL)	PEG-OH	1.78 ± 0.01	1.71 ± 0.02	0.65 ± 0.01	1.02 ± 0.01
	PEG-C16	9.61 ± 0.08	10.84 ± 0.06	6.29 ± 0.02	7.96 ± 0.01
	PEG-C18	9.42 ± 0.13	11.60 ± 0.01	9.81 ± 0.01	9.18 ± 0.01
	Myrj™ S40	20.81 ± 0.15	24.15 ± 0.06	16.76 ± 0.02	18.16 ± 0.01
	SA-PEG$_{100}$ GalNAc	0.00	3.47 ± 0.04	8.17 ± 0.03	16.63 ± 0.01
EXPERIMENTAL quantity in total NLC sample (µmol)	PEG-OH	5.01 ± 0.02	4.80 ± 0.06	1.84 ± 0.01	2.87 ± 0.01
	PEG-C16	27.02 ± 0.23	30.49 ± 0.17	17.69 ± 0.05	22.40 ± 0.01
	PEG-C18	26.49 ± 0.35	32.63 ± 0.01	27.60 ± 0.02	25.80 ± 0.01
	Myrj™ S40	58.52 ± 0.43	67.92 ± 0.16	47.13 ± 0.06	51.07 ± 0.02
	SA-PEG$_{100}$ GalNAc	0.00	3.25 ± 0.03	7.64 ± 0.02	15.55 ± 0.01
THEORETICAL quantity in total NLC sample (µmol)	PEG-OH	60.73	57.65	52.51	42.44
	PEG-C16	59.18	56.18	51.17	41.36
	PEG-C18	59.56	56.54	51.49	41.62
	Myrj™ S40	179.47	170.36	155.17	125.42
	SA-PEG$_{100}$ GalNAc	0.00	3.64	8.74	19.72
**EXPERIMENTAL molar % of incorporation**	PEG-OH	8.25 ± 0.03	8.34 ± 0.10	3.50 ± 0.01	6.77 ± 0.03
	PEG-C16	45.66 ± 0.38	54.28 ± 0.31	34.58 ± 0.09	54.15 ± 0.02
	PEG-C18	44.48 ± 0.59	57.73 ± 0.02	53.59 ± 0.03	61.99 ± 0.01
	**Myrj™ S40**	**32.61 ± 0.24**	**39.88 ± 0.09**	**30.37 ± 0.04**	**40.72 ± 0.01**
	**SA-PEG$_{100}$ GalNAc**	**0.00**	**89.52 ± 0.95**	**87.42 ± 0.28**	**78.89 ± 0.03**
**EXPERIMENTAL molar % in formulation**	**SA-PEG$_{100}$ GalNAc**	**0.0**	**4.6 ± 0.05**	**13.9 ± 0.04**	**23.3 ± 0.01**

From data of Table S1, the number of GalNac/particle can be calculated assuming all formulations display the same lipid core diameter (same composition of core lipids (oil, wax, lecithin)) (40 nm), and taking lipid core density equals to 1.05 g/cm^3. Calculations are detailed in Table 2.

Table S2. Calculation of the number of GalNac per particle.

		A	B	C	D
$\%_{mol}$ SA-PEG$_{100}$-GalNAc		**0**	**4.6**	**13.9**	**23.3**
SA-PEG$_{100}$-GalNAc number (total sample)		0	19.1 10^{17}	46.0 10^{17}	93.6 10^{17}
NLC lipid core diameter	(nm)	40	40	40	40
Volume of 1 particle	(cm^3)	3.35 10^{-17}	3.35 10^{-17}	3.35 10^{-17}	3.35 10^{-17}
Weight of 1 particle	(g)	3.52 10^{-17}	3.52 10^{-17}	3.52 10^{-17}	3.52 10^{-17}
NLC total sample mass	(g)	0.493	0.506	0.486	0.507
NLC number in total sample		14.0 10^{15}	14.4 10^{15}	13.8 10^{15}	14.4 10^{15}
**SA-PEG$_{100}$-GalNAc / NLC**		**0**	**135**	**333**	**650**

From the number of SA-PEG$_{100}$-GalNAc per particle, the ligand surface density can be deduced (Table S3). In this case, the hydrodynamic diameter of the particles issued from DLS measurements is used, assuming all ligands are located on the particle surface. From the ligand surface density, the distance between two neighbor ligands can be estimated, as the root square of the surface per 1 SA-PEG$_{100}$-GalNAc.

Table S3. Calculation of GalNac density on each type of particle.

		A	B	C	D
$\%_{mol}$ SA-PEG$_{100}$-GalNAc		**0**	**4.6**	**13.9**	**23.3**
SA-PEG$_{100}$-GalNAc / NLC		0	135	333	650
NLC hydrodynamic diameter	(nm)	42 ± 1	37 ± 1	52 ± 1	60 ± 1
NLC hydrodynamic surface	(nm^2)	5 489 ± 131	4 347 ± 117	8 626 ± 166	11 234 ± 187
**Surface / 1 SA-PEG$_{100}$-GalNAc**	**(nm^2)**	**0**	**32**	**26**	**17**
**Distance between 2 SA-PEG$_{100}$-GalNAc**	**(nm)**	**-**	**5.7**	**5.1**	**4.1**

Comparison with bibliographic data

The present results were compared with the bibliographic data from Morille et al. [1]. In this bibliographic study, two types of Gal ligands, DSPE-PEG$_{2000}$-Gal[1] and F108-Gal (Figure S6), were post-inserted at the surface of lipid nanocapsules investigated for gene therapy. These nanocapsules were composed of a liquid lipid core surrounded by a shell of amphiphilic surfactants and possessed structure and physico-chemical properties close to those of herein described NLC, though different. The number of saccharide units per particle was given in the paper, assuming all ligands were inserted at the particle surface. Therefore, comparison could be made in terms of ligand density between these literature data and ours, as reported in Table S4. To note that in our study we inserted GalNac ligands, whereas in the case of [1], it was Gal ligands.

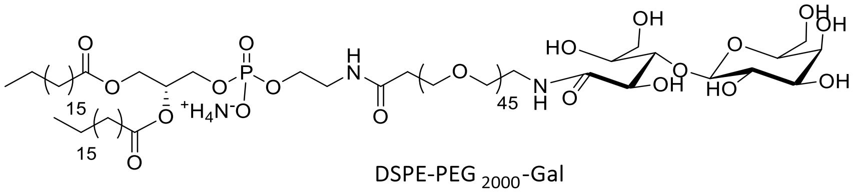

DSPE-PEG 2000-Gal

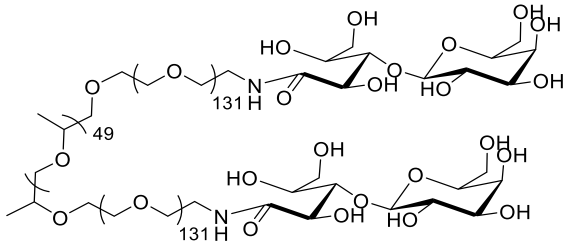

F108-Gal

Figure S6. Structures of DSPE-PEG$_{2000}$-Gal and F108-Gal inserted in lipid nanocapsules described in [1].

Table S4. Comparative data of ligand density between our study and that of Morille et al. [1].

	DPSE-PEG$_{2000}$-Gal (mM)			F108-Gal (mM)			SA-PEG$_{100}$-GalNAc (mM)		
	2	5	10	1	2	3	0.65 (B)	1.53 (C)	3.11 (D)
Ligands/particle	324	813	1 627	324	648	972	135	333	650
NP diameter (nm)	132	136	172	129	138	182	37	52	60
NP surface (nm^2)	54 739	58 107	92 941	52 279	59 828	104 062	4 347	8 626	11 234
**Surface / ligand (nm^2)**	**169**	**71**	**57**	**161**	**92**	**107**	**32**	**26**	**17**

The authors reported that DSPE-PEG$_{2000}$-Gal coating (up to 1,627 ligands/particle, 1 ligand/57 nm^2) did not induce significant effect on gene delivery in primary rat hepatocytes during transfection

[1] Here PEG$_{2000}$ refers to PEG with 2,000 g/mol molecular weight (about 40 ethylene(glycol) units).

studies, in comparison to unmodified nanocapsules. On the contrary, F108-Gal coated nanocapsules (up to 972 ligands/particle, 1 ligand/107 nm^2) strongly improved the transfection efficiency. Therefore, the results could not be directly correlated to the surface density (Table S4), and suggested a difference in sugar accessibility. Indeed the longer PEG chains in F108 compared to DSPE-PEG_{2000} could allow a suitable accessibility of terminal Gal units at the cell surface for multivalent interaction with the ASGPR.

References

1. Morille, M.; Passirani, C.; Letrou-Bonneval, E.; Benoit, J.P.; Pitard, B. Galactosylated DNA lipid nanocapsules for efficient hepatocyte targeting. *International Journal of Pharmaceutics* **2009**, 379, 293-300.
